# Non-Enzymatic Selective Detection of Histamine in Fishery Product Samples on Boron-Doped Diamond Electrodes

**DOI:** 10.3390/bios15080489

**Published:** 2025-07-29

**Authors:** Hiroshi Aoki, Risa Miyazaki, Yasuaki Einaga

**Affiliations:** 1 Environmental Management Research Institute, National Institute of Advanced Industrial Science and Technology (AIST), 16-1 Onogawa, Tsukuba 305-8569, Ibaraki, Japan; miyazaki.risa@aist.go.jp; 2 Department of Chemistry, Faculty of Science and Technology, Keio University, 3-14-1 Hiyoshi, Kohoku-ku, Yokohama 223-8522, Kanagawa, Japan

**Keywords:** histamine, food environment, electrochemical detection, fish samples, boron-doped diamond (BDD)

## Abstract

Histamine sensing that uses enzymatic reactions is the most common form of testing due to its selectivity for histamine. However, enzymes are difficult to store for long periods of time, and the inactivation of enzymes decreases the reliability of the results. In this study, we developed a novel, quick, and easily operated histamine sensing technique that takes advantage of the histamine redox reaction and does not require enzyme-based processes. Because the redox potential of histamine is relatively high, we used a boron-doped diamond (BDD) electrode that has a wide potential window. At pH 8.4, which is between the acidity constant of histamine and the isoelectric point of histidine, it was found that an oxygen-terminated BDD surface successfully detected histamine, both selectively and exclusively. Measurements of the sensor’s responses to extracts from fish meat samples that contained histamine at various concentrations revealed that the sensor responds linearly to the histamine concentration, thus allowing it to be used as a calibration curve. The sensor was used to measure histamine in another fish meat sample treated as an unknown sample, and the response was fitted to the calibration curve to perform an inverse estimation. When estimated in this way, the histamine concentration matched the certified value within the range of error. A more detailed examination showed that the sensor response was little affected by the histidine concentration in the sample. The detection limit was 20.9 ppm, and the linear response range was 0–150 ppm. This confirms that this sensing method can be used to measure standard histamine concentrations.

## 1. Introduction

To be guaranteed a safe and non-toxic diet, consumers need to be protected from food that has been contaminated. Keeping food safe requires management not only by the upstream food providers in the supply chain, but also through awareness on the part of downstream food consumers. Histamine poisoning has been a problem throughout history, and since it does not decompose, even when heated, it has been subject to regulation for many years [1,2,3]. Histamine-producing bacteria are always present in the natural environment, and histamine is produced when histidine decarboxylase breaks down histidine, an amino acid. Foods that contain high levels of histidine are more likely to cause histamine poisoning because histidine is converted into histamine when it is acted on by histamine-producing bacteria [1]. The UN’s Food and Agriculture Organization (FAO) requires that food products must not contain more than 100 ppm of histamine (averaged over multiple samples) or 200 ppm (in any individual sample) [2,4,5,6]. The European Commission Regulations have established that, in fishery products from histidine-rich fish (e.g., Scombridae, Clupeidae, and Engraulidae), the histamine limit should be 100 ppm (averaged over multiple samples) or 200 ppm (in any individual sample), and those for enzyme-treated fish products should be 200 ppm (averaged over multiple samples) or 400 ppm (in any individual sample) [7,8]. In the US, the Food and Drug Administration (FDA) has set a toxicity level of 50 ppm and a caution level at 500 ppm of histamine in any food [9]. For this reason, techniques for detecting histamine levels have been developed. The most widely used and officially accepted method for determining histamine in fishery-sourced food is a fluorometric method established by the Association of Official Analytical Chemists (AOAC) [10] or high-performance liquid chromatography (HPLC) [11,12], both of which require a lengthy pretreatment process for food samples.

Histamine sensing based on enzyme reactions has been the mainstream technique up to now due to its selectivity for histamine [13,14]. Sensing methods based on enzymatic reactions have several advantages. First, the specificity of the enzyme itself for the substrate being detected can contribute to the selectivity of the sensing results. Second, the sensing results can be obtained by converting them into redox reactions. This has the advantage that it is easy to construct detection systems based on electrochemical reactions, such as color reactions and sensor responses. Third, such enzymes can be synthesized on a large scale using microorganisms, which may lead to less expensive sensing methods in the future. Most enzyme-based histamine-sensing tests use histamine oxidase (HOX) or histamine dehydrogenase (HDH). The principle of this method is based on the generation of electrons in the oxidation reaction through which histamine is broken down by HOX or HDH into imidazole acetaldehyde. At this time, for example, the generated electrons can be accepted by a tetrazolium salt WST-8 [2-(2-methoxy-4-nitrophenyl)-3-(4-nitrophenyl)-5-(2,4-disulfophenyl)-2H-tetrazolium monosodium salt] in the presence of the electron mediator 1-methoxy PMS [1-methoxy-5-methylphenazinium methylsulfate]. This results in a color reaction in WST-8 that indicates the presence of histamine [15,16]. Similarly, the electrons can be collected by an electrode in the presence of a mediator, such as ferrocene [17], tetrathiafulvalene [18], and Os complexes [19,20], and they can be detected as the intensity of an electric current. Typical concentration ranges for enzyme-based histamine detection are reported to be of a 10^−6^ M (= 111.15 ppm) level or of a 10^−8^ M level with subsequent enhancement processes, including molecular imprinting and antibody- and nanoparticle-based technology [13,14]. These cover the ranges of histamine concentrations in foods in applications such as fish products (fresh, frozen, and canned products) and drinks (wine, milk, and juice) [13].

However, sensing methods that are based on enzymatic reactions have several disadvantages. The enzymatic reaction is usually slow, so it takes time to obtain the results. For example, a wait of up to 20 min is needed for the color reaction to develop [13,16]. Since enzymes are proteins and thus unstable, they need to be stored under ideal conditions to prevent inactivation. This makes it difficult to use them in environments that cause decreased enzyme activity. Since partially inactivated enzymes have a deleterious effect on measurement results, they must be used before they are inactivated, which means that they have expiration dates. This makes it difficult to keep a ready supply of fresh and immediately usable enzymes that can be employed in any environment. In brief, the chief drawbacks of enzymatic methods are the lack of long-term stability of the enzymes, the complex procedures required, and the long wait for results.

Boron-doped diamond (BDD) electrodes have a wide potential window and are known to be useful in non-enzymatic amino acid sensing [21]. BDD electrodes have a large overpotential for hydrogen and oxygen evolution and a low background current compared with other electrode materials. This is because the diamond surface, which is made of sp^3^ carbon, has very few sites on which molecules can be adsorbed [22,23]. Histamine, like other amino acids, has an oxidation potential of +1 V Ag/AgCl or more, so it is possible to observe the oxidation wave of histamine on a BDD electrode. However, considering that histamine is produced from the microbial reaction of histidine and that both histamine and histidine have the same imidazole group, histidine appears to mainly act as an interference substance during histamine detection [1]. Since both molecules have imidazole groups and, therefore, have almost the same oxidation potential under neutral aqueous conditions of around pH 7, ensuring histamine selectivity over histidine has been a challenge.

**Scheme 1:**
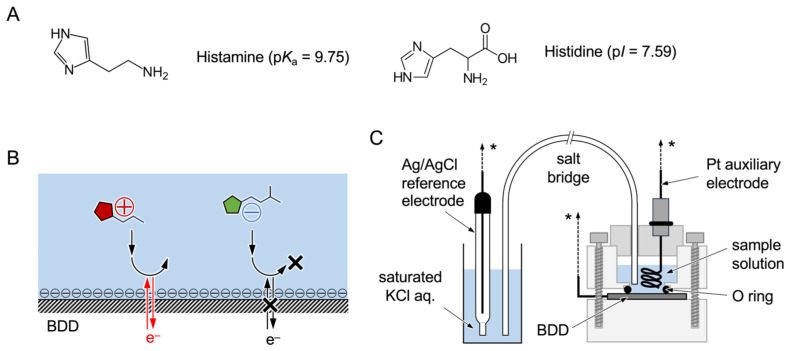
The chemical structures of histamine and histidine and their isoelectric points. (**A**) Scheme showing the working principle of this study. (**B**) In PBS solutions at pH 8.4, the O-BDD surface facilitates the redox reaction of histamine, which is positively charged, but it inhibits that of histidine, which is negatively charged, due to the electrostatic interaction. Electrochemical setup used in this study. (**C**) The electrochemical cell consists of Teflon molds placed on the BDD via an O-ring as a working electrode filled with 1 mL of the measurement sample solution with the Ag/AgCl reference and Pt auxiliary electrodes, connected to the electrochemical analyzer (shown as *).

In this study, we aimed to develop a simple technology for histamine sensing that is not based on an enzyme reaction or other special processes. The surface of a BDD electrode was oxidized to form oxygen termini that would provide greater selectivity for histamine than for histidine. The pH of the sample solution was also placed between the acidity constant of histamine (p*K*_a_) and the isoelectric point of histidine (p*I*) (Scheme 1A). We aimed, using these two techniques, to selectively detect the oxidation wave of histamine. When the oxidation reaction of histamine and histidine was observed with the pH of the sample solution set at 8.4, we found that the oxidation reaction of histamine began at a potential of about 0.1 V lower than that of histidine. We surmised that this was due to the electrostatic relationship between the BDD surface and histamine or histidine as determined by the values of pH, p*K*_a_, and p*I*, where the oxygen terminus on the BDD surface and histidine have negative charges, while histamine has a positive charge (Scheme 1B). This selectivity was also observed in measurements using a sample of fish meat. The histamine concentration vs. sensor response was plotted by measuring a fish meat sample extract with a known histamine concentration. A highly linear calibration curve was obtained over the range of 0–150 ppm (*R*^2^ = 0.968, with a detection limit of 20.9 ppm). The histamine concentration was estimated from the sensor response in another fish meat sample extract using this calibration curve. It was found that the histamine concentration in the sample could be estimated with high accuracy. To the best of our knowledge, this method is the first to use a BDD electrode that can selectively detect histamine in a practical concentration range in real samples without using enzymes. We also anticipate it to be of use as a new, rapid, and easily operated histamine detection technique.

## 2. Materials and Methods

### 2.1. Reagents

The canned fish meat samples (tuna) used in this study were purchased from Fera Science Ltd. (Sand Hutton, York, UK), and they were originally prepared as quality control materials for the proficiency test. The histamine concentrations in the samples were certified based on HPLC to be 273 ± 19 ppm (Sample A; Product code FCAL10-SEA7QC, Material code T27390QC) and 27.3 ± 2.7 ppm (Sample B; Product code FCAL11-SEA7QC, Material code T27381QC) according to the information provided by the manufacturer (Scheme S1) [24,25]. The purchased samples were fractionated into 1 g aliquots and stored in a refrigerator at –20 °C until use. Histamine, histidine, Na_2_HPO_4_, and 10× phosphate-buffered saline (10× PBS) were purchased from Fujifilm-Wako Chemical Corporation (Tokyo, Japan). Then, 1× PBS was prepared from 10× PBS through tenfold dilution. All of the aqueous solutions were made up with deionized and charcoal-treated water (specified resistance > 18.2 MΩ cm), which was obtained using a Milli-Q reagent-grade water system (Merck-Millipore; Bedford, MA, USA).

### 2.2. Preparation of BDD Electrodes

BDD electrodes were prepared on Si(100) wafers based on a microwave-plasma-assisted chemical vapor deposition system (ASTex-5400; Cornes Technologies, Tokyo, Japan), according to a previously reported procedure [26,27]. Acetone and trimethyl borate were used as the carbon and boron sources, respectively, with a B/C atomic ratio of 1%. The prepared BDD film was confirmed using Raman spectroscopy (Figure S1). The thickness of the BDD film was obtained to be about 40 µm with a deposition time of 10 h and a microwave power of 5 kW.

### 2.3. Electrochemical Measurements of BDD Electrodes

The electrochemical measurements were carried out using an ALS-760C electrochemical analyzer (Bioanalytical Systems (BAS), Tokyo, Japan) based on a three-electrode configuration consisting of a Pt auxiliary electrode, a Ag/AgCl reference electrode, and the BDD electrode as the working electrode. The reference electrode was equipped with a salt bridge (internal solution: 3 M KCl) (Scheme 1C). The area of the working electrode was 0.28 cm^2^ (circular, with a diameter of 0.6 cm). The BDD electrodes were electrochemically cleaned and activated through chronoamperometry (CA) by applying a potential of −3.0 V for 5 min (cathodic reduction) and then a potential of +3.0 V for 5 min (anodic oxidation) in 1× PBS before use. The anodic oxidation resulted in an oxygen layer on the BDD surface [28]. Electrochemical measurements for histamine, histidine, and the fish samples were conducted using linear sweep voltammetry (LSV), where the potential was scanned from 0 to +2.0 V and back to 0 V with a scan rate of 0.1 V s^−1^.

### 2.4. Preparation of Sample Solutions for Electrochemical Measurements

The electrochemical measurements were performed at pH 7.4 and pH 8.4. Here, pH 7.4 was chosen as the typical pH of foods, and pH 8.4 was chosen according to the p*K*_a_ (or p*I*) for histamine and histidine. Sample solutions were prepared as mixtures of solutions of 50 mM histamine, 50 mM histidine, fish samples, and 0.5 M Na_2_HPO_4_. A mixture of 1:2 (*w*/*w*) of the fish meat sample and 0.5 M Na_2_HPO_4_ was centrifuged at 25 °C and 10,000 rpm for 10 min. The obtained supernatant (pH 8.4) was used as the fish extract in this study. The histamine- and/or histidine-containing fish extract was prepared as described in Scheme S2. The fish extract sample was prepared as a 1:9 mixture of the fish extract and other solutions.

### 2.5. Inverse Estimation

In this study, the concentrations of histamine in the samples were predicted through inverse estimation via electrochemical analysis. When a calibration curve *y* = *a* + *bx* is obtained for a concentration (*x*)-signal (*y*) plots with *n* data, if the value of the signal (*y*_A_) is provided as an average of *m* observations from the analysis, the corresponding value of the concentration (*x*_A_) and its standard deviation ($\sigma_{x_{A}}$) can be calculated using inverse estimation as follows [29]:

*x*_A_ = (*y*_A_ − *a*)/*b* (1)

(2) $$\sigma_{x_{A}}=\frac{\sigma_{y/x}}{b}\sqrt{\frac{1}{m}+\frac{1}{n}+\frac{{\left(y_{A}-\bar{y}\right)}^{2}}{b^{2}\sum_{i}{\left(x_{i}-\bar{x}\right)}^{2}}}$$

where $\sigma_{y/x}=\sqrt{\frac{\sum_{i}{\left(y_{i}-{\hat{y}}_{i}\right)}^{2}}{n-2}}$ (${\hat{y}}_{i}$ was calculated from *a* + *bx_i_*), $\bar{x}$ is the average of *x_i_*, and $\bar{y}$ is the average of *y_i_*.

## 3. Results and Discussion

### 3.1. Electrochemical Responses to Histamine and Histidine in PBS

The oxidation potential of histamine is generally relatively high, and it is at around +1 V vs. Ag/AgCl or higher [30,31]. The BDD electrode has an extremely wide potential window from approximately −2 V to +2 V, and it is characterized by a very low charging current [22]. Therefore, we used a BDD electrode in this study to observe the oxidation reaction of histamine.

**Figure 1:**
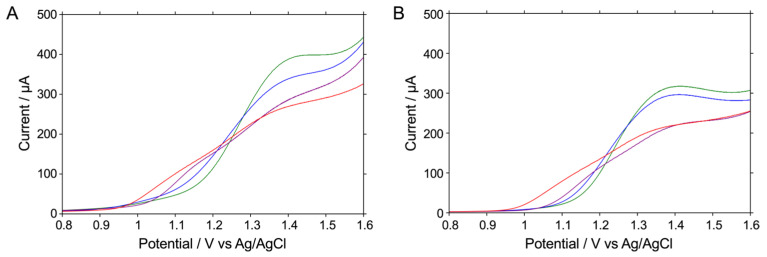
LSV readings for the oxidative reaction of 1 mM histamine or 1 mM histidine in 20 mM Na_2_HPO_4_ at pH 8.4 (red or blue) and in 1× PBS at pH 7.4 (purple or green), respectively, on an H-BDD electrode (**A**) or an O-BDD electrode (**B**). All of the LSV readings were recorded by scanning the potential from 0.8 to 1.6 V at a scan rate of 0.1 V s^−1^ at room temperature.

Histamine and histidine, which are the most likely contaminants and sources of histamine in microbial reactions, have an imidazole moiety. Both histamine and histidine can show oxidation waves at a similar potential [32]. Figure 1A shows the LSV results that vary according to the concentrations of histamine or histidine in 1× PBS at pH 7.4 on an as-prepared BDD (hydrogen-terminated BDD, H-BDD) electrode. Histamine began to be oxidized at around +1.05 V, and a broad oxidation wave was observed beyond +1.4 V. Histidine, on the other hand, started to be oxidized at around +1.15 V, although the oxidation waves were smaller than those for histamine at the same concentrations, and larger oxidation waves were observed at around +1.4 V.

In our experiments, the higher the histamine concentration, the greater the oxidation wave observed, but it was found that the concentration and the oxidation current value were clearly not proportional at around +1.4 V. This suggests that a polymerization reaction may occur at around +1.4 V [33]. Molecules with imidazole moieties, including histamine and histidine, are known to polymerize when higher potentials are applied to the electrode [33,34]. In fact, after the first electronic reaction occurs at the low-potential side, electropolymerization proceeds, forming a polymer that is easily adsorbed on the electrode surface, inhibiting the electrode reaction [31]. Therefore, it seems probable that the oxidation current value is less likely to reflect the histamine concentration. On the other hand, it is known that a one-electron reaction occurs at around +1.0 V, where electropolymerization does not occur, and that the oxidation current value is proportional to the histamine concentration [33]. Therefore, in the following study, we decided to focus on the current value at a potential of around +1.0 V instead of those at higher potentials or the peak potential.

Figure 1B shows the LSV-measured concentration dependence of histamine or histidine at various concentrations measured on the anodized BDD (oxygen-terminated BDD, O-BDD) electrode in 1× PBS at pH 7.4. Histamine started to be oxidized at around +1.05 V, while histidine started to be oxidized at around +1.15 V. In other words, it was observed that histidine is less readily oxidized than histamine and requires a higher potential to be oxidized. This indicates that the anodization of the BDD surface resulted in oxygen termination and a negative charge, whereas in a solution at pH 7.4, histamine (p*K*_a_ = 9.75 [35]) is positively charged and histidine (p*I* = 7.59; i.e., the average of p*K*_aR_ = 6.00 and p*K*_a2_ = 9.17 [36]) is almost electrically neutral, facilitating the oxidation of histamine (Scheme 1A,B and Schemes S3). In other words, at +1.05 V, almost no oxidation current was observed for histidine, and a concentration-dependent change in the oxidation current was observed that was selective for histamine. This investigation, therefore, shows an expectation that by using an O-BDD electrode and optimizing the pH of the solution, histamine can be more selectively detected without relying on enzymatic reactions.

### 3.2. pH Dependence of Electrochemical Responses to Histamine and Histidine

We examined the selectivity of histamine detection as a function of the pH of the solution. The oxidation reactions of 1 mM histamine and 1 mM histidine at pH 7.4 and pH 8.4 were compared by measuring the LSV readings when using the H-BDD and O-BDD electrodes. According to Figure 1A, which shows the LSV measurements with the H-BDD electrode, the difference in the oxidation potentials for histamine and histidine was 100 mV at pH 7.4. When these measurements were performed at pH 8.4, both of the oxidation potentials for histamine and histidine shifted to the lower-potential side. As a result, the difference in the oxidation potentials remained almost unchanged at 100 mV. Figure 1B, showing the LSV when using the O-BDD electrode, shows the difference in oxidation potentials to be at most about 100 mV at pH 7.4. However, in LSV measured at pH 8.4, the oxidation potential for histidine shifted to the lower-potential side by about 20 mV, while that for histamine shifted to the lower-potential side by about 100 mV. As a result, the difference in oxidation potentials widened to 180 mV. In short, the difference in oxidation potentials was wider with the O-BDD than with the H-BDD, suggesting that the O-BDD electrode gives better selectivity for histamine detection. The reason for this is that in a solution at pH 8.4, histamine is still positively charged, but more histidine is negatively charged than at pH 7.4. This enhances the selectivity of histamine, and the effect of the negative charge on the electrode surface is large with the O-BDD, as shown in Scheme 1B. Therefore, we concluded that the combination of the use of solutions at pH 8.4 and the O-BDD electrode is better for the selective detection of histamine.

**Figure 2:**
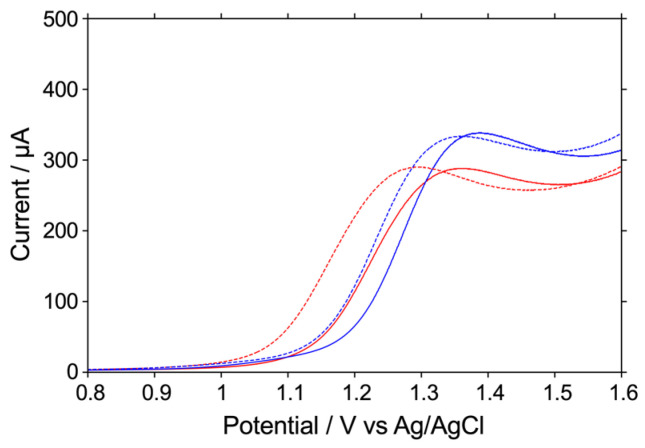
LSV readings for the oxidative reaction of 1 mM histamine or 1 mM histidine in 0.5 M Na_2_HPO_4_ at pH 7.4 (red or blue; solid) or pH 8.4 (red or blue; dashed), respectively, on an O-BDD electrode. All of the LSV readings were recorded by scanning the potential from 0.8 to 1.6 V at a scan rate of 0.1 V s^−1^ at room temperature.

In these experiments, PBS was used as a realistic buffer solution for the study at pH 7.4, and 20 mM phosphate buffer was used as a buffer solution with almost the same salt concentration as PBS for the study at pH 8.4. However, real samples for human consumption often have high salt concentrations. We concluded that high concentrations of salt were likely used to improve the long-term storage stability of foods. Therefore, we believed that it was necessary to conduct the same study at high salt concentrations. In fact, for the fish meat sample used in the next section, it was necessary to use a phosphate buffer with a high salt concentration of 0.5 M to adjust the pH. Figure 2 shows the LSV measurements of the oxidation reactions of 1 mM histamine and 1 mM histidine at pH 7.4 and pH 8.4 using an O-BDD electrode. As a result, as in the case of low salt concentrations, the shift in oxidation potential when the pH was changed from 7.4 to 8.4 was clearly larger for histamine (about 100 mV) than for histidine (about 50 mV), and the difference in oxidation potential increased. In other words, it was revealed that the use of an O-BDD electrode is advantageous for the selective detection of histamine, regardless of the salt concentration.

In the results shown in Figure 1, it is also seen that the background current in the LSV measurements was smaller with the O-BDD electrode than with the H-BDD electrode. In general, the background current tended to be smaller with the H-BDD electrode because there was less charge on the electrode surface. Although the reasons for the background current being smaller with the O-BDD electrode need further investigation, we also concluded here that the O-BDD electrode is preferable from this point of view. In our subsequent experiments, the O-BDD electrode was used at pH 8.4 to detect histamine.

### 3.3. Preparation of Fish Meat Sample Extracts

The fish meat samples used in this study were materials for proficiency testing in food analysis, and the histamine concentration in the samples was certified by the manufacturer [24]. The fish meat samples were in the form of a uniform paste that was easily dispersed in the electrolyte solutions. We used the simple pretreatment method described in the Materials and Methods section, in which the fish meat samples were dispersed in 0.5 M phosphate buffer at pH 8.4 and then centrifuged to precipitate the residue. The resulting supernatant was used to prepare the fish extract samples, which were then subjected to electrochemical measurements.

**Figure 3:**
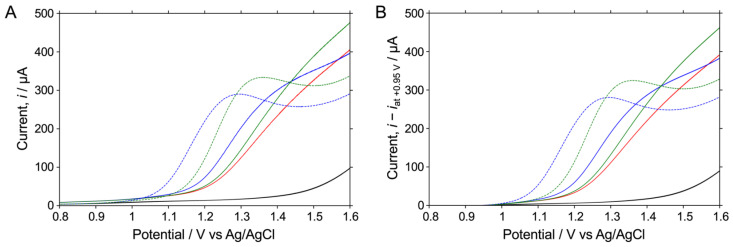
LSV readings for the oxidative reaction of 1 mM histamine or 1 mM histidine in 0.5 M Na_2_HPO_4_ at pH 8.4 (blue or green; dashed) or in the fish extract sample of 0.5 M Na_2_HPO_4_ at pH 8.4 (blue or green; solid), respectively, on an O-BDD electrode. (**A**) LSV readings in 0.5 M Na_2_HPO_4_ at pH 8.5 and in the fish extract sample in 0.5 M Na_2_HPO_4_ at pH 8.4 are also shown for reference (black). Current values in each LSV in (**B**) are shown as the current minus that at +0.95 V in each LSV in (**A**). All of the LSV readings were recorded by scanning the potential from 0.8 to 1.6 V at a scan rate of 0.1 V s^−1^ at room temperature.

Figure 3A shows the LSV readings (red solid line) obtained in the fish extract sample prepared through extraction from a fish meat sample (Sample A; Material code T27390QC). Comparing the LSV readings in phosphate buffer (black solid line) with those in phosphate buffer containing 1 mM histamine (blue dotted line) and 1 mM histidine (green dotted line) showed that the oxidation potential significantly shifted to the higher potential side. This appears to be because various components, such as proteins and fats contained in the fish meat sample, interfere with the electrode reaction on the electrode surface. In this study, when 1 mM histamine was present in a fish extract sample (blue solid line), the current value began to increase from around +1.1 V, indicating that the observed oxidation current value had increased due to the higher histamine concentration in the sample. On the other hand, in the LSV readings for the fish extract sample containing 1 mM histidine (green solid line), the current value was also higher than with the fish extract sample alone, but the potential at which the increase began had shifted to the higher potential side around +1.2 V. In brief, it was found that between +1.1 V and +1.2 V, the current value derived from histamine could be selectively detected.

On the other hand, Figure 3A shows that the baseline current value varies according to the measurement solution. Therefore, we believed that it would be possible to compare LSVs more accurately by using the current value obtained by subtracting the current value at +0.95 V from the observed current value as the baseline, as shown in Figure 3B. In the following sections, we discuss the results considering the current value obtained by subtracting the baseline current value from the sensor response.

### 3.4. Electrochemical Responses to Histamine and Histidine in Fish Meat Sample Extracts

**Figure 4:**
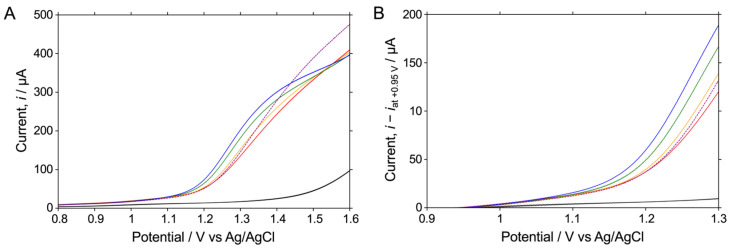
LSV readings for the oxidative reaction measured in the fish extract sample of 0.5 M Na_2_HPO_4_ at pH 8.4 containing no other substances, 0.1 mM histamine, 0.5 mM histamine, 1 mM histamine (red, orange, green, or blue; solid), or 1 mM histidine (purple; dashed) on an O-BDD electrode. (**A**) LSV measurements in 0.5 M Na_2_HPO_4_ at pH 8.4 are also shown for reference (black). The current values in each LSV in (**B**) are shown as the current minus that at +0.95 V in each LSV in (**A**), shown from +0.9 V to +1.3 V. All of the LSVs were recorded by scanning the potential from 0.8 to 1.6 V at a scan rate of 0.1 V s^−1^ at room temperature.

In the previous section, we found that the histamine-derived current value could be selectively detected at potentials of between +1.1 V and +1.2 V. We then investigated the histamine concentration dependence of the sensor response in fish extract samples. Figure 4A shows the LSV readings obtained in fish extract samples containing 0.1 mM–1 mM histamine and in phosphate buffer. Compared with the histamine-concentration-dependent current changes in PBS (Figure 1B), the change in current in the fish extract sample was narrower; however, a significantly histamine-concentration-dependent sensor response was observed. Figure 4B shows an enlarged view of the response from +1.1 V to +1.2 V. It is clearly shown that in this potential range, the current value, which is the sensor response, rises with increasing histamine concentration. The current value for 1 mM histidine was also similar to that for 0 mM histamine, with almost no sensor response being observed. The sensor response to the histamine and histidine concentrations was next examined in detail by evaluating the sensor response obtained by subtracting the current value at +0.95 V from the current value at +1.15 V.

**Figure 5:**
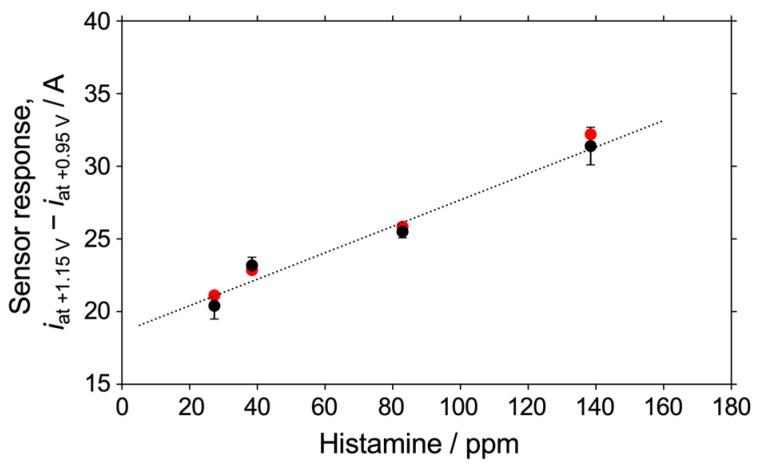
Plots of values of the current at +1.15 V minus that at +0.95 V in LSV readings measured in a fish extract sample (black plots) and in one containing 1 mM histidine (red plots). Other conditions are the same as those listed in the caption of Figure 4.

As a practical application, we tried to establish a calibration curve for the fish product. An investigation in standard solutions might not reflect practical applications in real samples. Figure 5 shows the dependence of the sensor response on the histamine concentration in fish extract samples extracted with 0.5 M phosphate buffer at pH 8.4. The horizontal axis shows the histamine concentration in ppm. Although the measured sensor responses were somewhat variable, a highly linear sensor response was observed between 0 ppm and 150 ppm, indicating that the oxidative currents in LSV readings are related to the redox reaction of free histamine in the samples. The regression line derived from these results is as follows:

*y* = 9.10 × 10^−8^ *x* + 1.86 × 10^−5^ (the correlation coefficient, *R*^2^ = 0.968) (3)

where *y* [A] indicates the sensor response, and *x* [ppm] indicates the histamine concentration. The detection limit was derived as 20.9 ppm, as calculated from an inverse regression using the values of the y-intercept and its standard deviation in Equation (3) (S/N = 3.0). The variation in the sensor response is likely due to the simple extraction method used in this study, which is based on stirring and precipitation. However, the highly linear response, in spite of our simple method, clearly indicates the significant potential of this method for application to real samples.

We also investigated the effect of histidine concentration on the sensor response. Figure 5 shows the sensor response (red plots) in fish extract samples containing 1 mM histidine and various concentrations of histamine (black plots). It was found that the response to histidine was well within the error of the response to histamine. This demonstrated the ability of this detection method to quantify the histamine concentration in fish extract samples with good linearity, even in the presence of 1 mM histidine.

### 3.5. Quantification of Histamine Concentrations in Different Samples

We verified whether unknown histamine concentrations could be quantified by using the regression line obtained in Equation (3) as a calibration curve. A fish meat sample from a different lot from those used to obtain the calibration curve was selected to test this. Electrochemical measurements were performed to obtain the sensor response of the BDD electrode, and the histamine concentration was calculated from the sensor response using inverse estimation and compared with the certified histamine concentration value of the fish meat sample. The sensor response obtained from the fish extract sample (a) prepared through extraction from the fish meat sample (Sample B; Material code T27381QC) was inversely estimated using the calibration curve. The result was 5.24 ± 9.79 ppm (*n* = 3), and the manufacturer-certified histamine concentration was 2.73 ± 0.27 ppm. The histamine concentrations of the samples (b, c, d, e, f) prepared by adding 0.1, 0.2, 0.3, 0.5, and 1 mM histamine to the fish extract sample (a) were similarly estimated. The values estimated using the calibration curve and the values obtained from the certified values are summarized in Table 1. The estimated values of these samples were all consistent with the corresponding certified histamine concentrations within the error range, and the histamine concentrations of the samples were very close to each other. Based on this, the method developed in this study is capable of measuring the histamine concentration in fish meat samples of unknown concentrations. This is anticipated to make a significant contribution as a rapid and easily applied method of quantifying histamine in actual food samples.

### 3.6. Limitations and Advantages of the Technique Developed in This Study

In this method, the electrochemical oxidation reaction of histamine at the BDD electrode is used as the sensor response. The prepared BDD electrode was repeatedly used. The BDD electrode showed sufficient stability during the study. In addition, the BDD electrode was electrochemically cleaned and activated by CA to obtain a good reproducibility.

We focused here on histidine. Histamine is most likely to be an interfering substance for histamine because histamine is produced from histidine as a result of bacterial reactions. We enhanced the histamine selectivity by selecting the pH of the measurement solution and O-BDD. We also succeeded in quantifying histamine from the sensor response at the potential where the currents for histidine were extremely low. The fish meat samples used in this study were ordinary canned fish, for which the histamine concentration was certified. The histamine concentration estimated using this method and the histamine concentration obtained from the certified value matched very closely. However, in some samples, other amino acids and other biological substances may cause interference. In addition to histidine, for example, tyrosine and cysteine are known to cause redox reactions at BDD electrodes [37]. Therefore, if we try to quantify the histamine concentration in a sample that contains large amounts of these amino acids, we might need to overcome new challenges. For example, we found that the method was applicable to fish sauces, including nam pla (a Thai fish sauce), nuoc mam (a Vietnamese fish sauce), and shottsuru (a Japanese fish sauce). However, we also found that some substances in the sauces might inhibit histamine sensing and needed a 50-fold or 100-fold dilution to measure the histamine in the sauces. Such dilution makes it difficult to measure histamine. The difficulty depends on the food samples. We would like to continue to investigate whether the calibration curve obtained in this study can be applied to samples other than fish meat.

An example of a conventional technique that does not use enzymes is a histamine detection technique using a copper phosphate electrode with a Nafion membrane fixed to the surface [38]. This technique focuses on the negative charge of Nafion, and it is similar to our technique, which focuses on negative charges on the BDD surface. However, Nafion membranes are prone to tearing and tend to absorb many chemical species, which limits their long-term use. The BDD electrode in this study is a robust electrode due to the nature of diamond electrodes, and even if the surface becomes dirty, it can always be cleaned using electrolytic polishing. This gives it a significant advantage over conventional techniques.

At food factories and other food management sites, speed is necessary to complete safety evaluations of the quality of large quantities of products. By using the method developed in this study, anyone can quickly and easily quantify histamine concentrations at any time and anywhere. It does not require the use of highly specialized equipment such as HPLC apparatuses, and unlike detection methods that use enzymes, there is no need to worry about enzyme deterioration. It is possible to expand the scope of application to not only the batch testing of foods but also the continuous monitoring of histamine concentrations, for example, to be able to issue real-time alerts.

## 4. Conclusions

In this study, we succeeded in developing a highly selective quantitative detection sensor for histamine in actual food samples by focusing on the histamine oxidation wave at a given pH under alkaline conditions using an O-BDD electrode. Histamine has a positive charge, and histidine has a negative charge under alkaline conditions. So, histamine is selectively oxidized on the electrode’s surface due to electrostatic interaction with the negative charge on the O-BDD electrode’s surface. The sensor response was found to respond linearly to histamine concentrations over the range of 0–150 ppm, with a detection limit of 20.9 ppm. Using the obtained calibration curve, we estimated the concentrations of histamine in samples other than those used for calibration. We found that the estimated value and the certified value showed good consistency within the range of error.

There have been many examples of electrochemical sensors for histamine, but most of them have achieved selectivity and sensor response by using histamine-degrading enzymes. Some methods not based on enzymes use special electrode materials or a combination of multiple materials other than electrodes, but these are complicated and require specialized skills. To the best of our knowledge, our method is the first study of a simple method that achieves selectivity for histamine and sensor response using only an O-BDD electrode and by adjusting the pH of the sample solutions. Our method also allows rapid measurement because, unlike in enzyme-based methods, the sensors respond instantly. We believe that this method has considerable potential for use as a standard, rapid, and simple method for histamine quantification at food processing and storage sites.

## Figures and Tables

**Table 1:** A comparison between the concentrations of histamine in food samples certified by the manufacturer and those obtained via electrochemical BDD electrode responses.

Sample	Histamine Derived from the BDD Responses [ppm]	Histamine Certified by the Manufacture [ppm]	Recovery Rate [%]	Relative Standard Deviation
a	5.24 ± 9.79	2.73 ± 0.27	(192)	(1.87)
b	15.28 ± 9.36	13.85 ± 0.27	110	0.61
c	22.02 ± 9.10	24.97 ± 0.27	88.2	0.41
d	34.03 ± 8.70	36.09 ± 0.27	94.3	0.26
e	52.66 ± 8.29	58.31 ± 0.27	90.3	0.16
f	113.71 ± 8.89	113.88 ± 0.27	99.9	0.078

## Data Availability

The datasets generated and/or analyzed during the current study are available from the corresponding authors upon reasonable request.

## Supplementary Materials

The online version contains supplementary material available at https://www.mdpi.com/article/10.3390/bios15080489/s1, Scheme S1: Detailed calculation of the concentrations of histamine in Sample A and Sample B; Scheme S2: The methods for preparation of all of the solutions in this study; Scheme S3: Chemical species of histamine and histidine as a function of pH; Figure S1: Raman spectrum of the BBD film on the electrode. A peak at 1300 cm^−1^ denotes sp^3^ carbon bands. The two peaks observed at around 500 and 1200 cm^−1^ indicate boron doping in the diamond structure. No peak was observed at around 1600 cm^−1^ related to sp^2^ carbon, confirming the successful preparation of the BDD film.
