# Supplementary material for: Non-Enzymatic Selective Detection of Histamine in Fishery Product Samples on Boron-Doped Diamond Electrodes
Source: Biosensors (Basel). 2025 Jul 29;15(8):489. doi: 10.3390/bios15080489 (PMC12385128; doi:10.3390/bios15080489)
Supplement: Supplementary file 1 [file biosensors-15-00489-s001.zip › biosensors-3745945-supplementary.pdf]

biosensors

MDPI

Article

Non-Enzymatic Selective Detection of Histamine in Fishery Product Samples on Boron-Doped Diamond Electrodes

Hiroshi Aoki [1,2,*], Risa Miyazaki [1,2] and Yasuaki Einaga [2,*]

[1] Environmental Management Research Institute, National Institute of Advanced Industrial Science and Technology (AIST), 16-1 Onogawa, Tsukuba 305-8569, Ibaraki, Japan; miyazaki.risa@aist.go.jp
[2] Department of Chemistry, Faculty of Science and Technology, Keio University, 3-14-1 Hiyoshi, Kohoku-ku, Yokohama 223-8522, Kanagawa, Japan
* Correspondence: aoki-h@aist.go.jp (H.A.); einaga@chem.keio.ac.jp (Y.E.)

Supplementary Materials

List of captions

Scheme S1. Detailed calculation of the concentrations of histamine in Sample A and Sample B.

Scheme S2. The methods for preparation of all the solutions in this study.

Scheme S3. Chemical species of histamine and histidine as a function of pH.

Figure S1. Raman spectrum of the BBD film on the electrode. A peak at 1,300 cm^{-1} denotes sp^3 carbon bands. Two peaks observed at around 500 and 1,200 cm^{-1} indicate boron doping in the diamond structure. No peak was observed at around 1,600 cm^{-1} related to sp^2 carbon, confirming the successfully preparation of the BDD film.

Scheme S1. Detailed calculation of the concentrations of histamine in Sample A and Sample B.

Fish sample name	Assigned value, x_a [ppm]*1, *2	Range for $\vert z \vert \leq 2$ ($z_{small} - z_{large}$)*3	Standard deviation, σ_p*4	Calculated histamine concentration [ppm]
Sample A	273	235 – 310	19	273 ± 19
Sample B	27.3	22.0 – 32.7	2.7	27.3 ± 2.7

*1 The values of the histamine concentration originally are shown in a unit of [mg kg^{-1}] in the manufacture's data sheets [1] Here, the unit was converted to [ppm].

*2 The assigned value is the average value of the histamine concentration measured in the proficiency test performed by the manufacture for the corresponding fish sample.

*3 A z-score is defined in the manufacture's instruction [2] as a deviation ($x - x_a$) divided by the standard deviation (σ_p) for the histamine concentration measured in the proficiency test, $z = (x - x_a)/\sigma_p$.

*4 The histamine concentration of the fish sample was calculated as follows. According to the definition of a z-score, the standard deviation, σ_p, can be derived as ($x_a - z_{small}$)/2 or ($z_{large} - x_a$)/2.

Scheme S2. The methods for preparation of all the solutions in this study.

(1) Fish extract, 2 mL
- Fish extract 200 µL
- 0.5 M Na_2HPO_4 1,800 µL

(2) Fish extract + 1 mM histamine, 2 mL
- 50 mM histamine 40 µL
- Fish extract 200 µL
- 0.5 M Na_2HPO_4 1,760 µL

(3) Fish extract + 1 mM histamine + 0.1 mM histidine, 2 mL
- 50 mM histamine 40 µL
- 50 mM histamine 4 µL
- Fish extract 200 µL
- 0.5 M Na_2HPO_4 1,756 µL

(4) Fish extract + 1 mM histamine + 0.5 mM histidine, 2 mL
- 50 mM histamine 40 µL
- 50 mM histamine 20 µL
- Fish extract 200 µL
- 0.5 M Na_2HPO_4 1,740 µL

(5) Fish extract + 1 mM histamine + 1 mM histidine, 2 mL
- 50 mM histamine 40 µL
- 50 mM histamine 40 µL
- Fish extract 200 µL
- 0.5 M Na_2HPO_4 1,720 µL

(6) Fish extract + 0.5 mM histamine, 2 mL
- 50 mM histamine 20 µL
- Fish extract 200 µL
- 0.5 M Na_2HPO_4 1,780 µL

(7) Fish extract + 0.5 mM histamine + 0.1 mM histidine, 2 mL
- 50 mM histamine 20 µL
- 50 mM histidine 4 µL
- Fish extract 200 µL
- 0.5 M Na_2HPO_4 1,776 µL

(8) Fish extract + 0.5 mM histamine + 0.5 mM histidine, 2 mL
- 50 mM histamine 20 µL
- 50 mM histidine 20 µL
- Fish extract 200 µL
- 0.5 M Na_2HPO_4 1,760 µL

(9) Fish extract + 0.5 mM histamine + 1 mM histidine, 2 mL
- 50 mM histamine 20 µL
- 50 mM histidine 40 µL
- Fish extract 200 µL
- 0.5 M Na_2HPO_4 1,720 µL

(10) Fish extract + 0.1 mM histamine, 2 mL
- 50 mM histamine 4 µL

- Fish extract 200 μL
- 0.5 M Na_2HPO_4 1,796 μL

(11) Fish extract + 0.1 mM histamine + 0.1 mM histidine, 2 mL

- 50 mM histamine 4 μL
- 50 mM histidine 4 μL
- Fish extract 200 μL
- 0.5 M Na_2HPO_4 1,792 μL

(12) Fish extract + 0.1 mM histamine + 0.5 mM histidine, 2 mL

- 50 mM histamine 4 μL
- 50 mM histidine 20 μL
- Fish extract 200 μL
- 0.5 M Na_2HPO_4 1,776 μL

(13) Fish extract + 0.1 mM histamine + 1 mM histidine, 2 mL

- 50 mM histamine 4 μL
- 50 mM histidine 40 μL
- Fish extract 200 μL
- 0.5 M Na_2HPO_4 1,756 μL

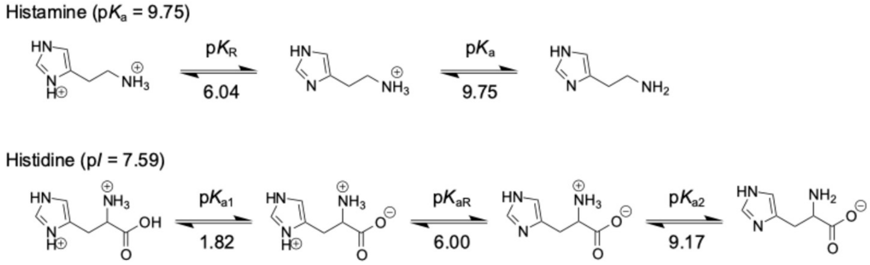

Scheme 3. Chemical species of histamine and histidine as a function of pH.

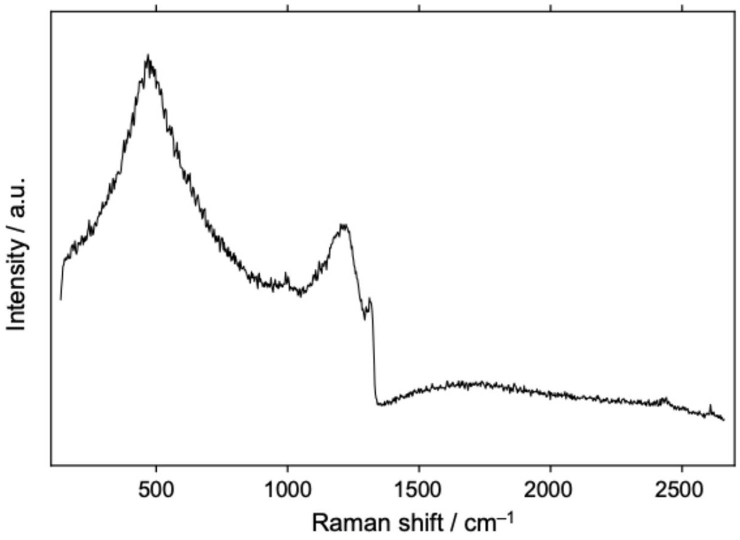

Figure S1. Raman spectrum of the BBD film on the electrode. A peak at 1,300 cm^{-1} denotes sp^3 carbon bands. Two peaks observed at around 500 and 1,200 cm^{-1} indicate boron doping in the diamond structure. No peak was observed at around 1,600 cm^{-1} related to sp^2 carbon, confirming the successfully preparation of the BDD film.

References

1. Fera, Fera Science Ltd, Part 1 - Common Principles. In *Protocol for Proficiency Testing Schemes*, 8 ed.; 2023. https://fapas.com/sites/default/files/2023-02/FeraPTSprotocol_pt1_common_v8_Jan2023_0.pdf
2. Fera, Fera Science Ltd, Part 2 - Fapas Food Chemistry scheme. In *Protocol for Proficiency Testing Schemes*, 6 ed.; 2023. https://fapas.com/sites/default/files/2023-02/FeraPTSprotocol_pt2_foodChem_v6_January2023.pdf
